# Effects of different dosages of Sodium-glucose cotransporter 2 inhibitors on glucose level change in patients with type 2 diabetes stratified by HbA1c and renal function: a systematic review and meta-analysis

**DOI:** 10.3389/fendo.2026.1785329

**Published:** 2026-03-02

**Authors:** Ruitong Xiong, Yucheng Yang, Yuxiu Li, Huabing Zhang

**Affiliations:** 1 Department of Endocrinology, NHC Key Laboratory of Endocrinology, Peking Union Medical College Hospital, Peking Union Medical College, Chinese Academy of Medical Sciences, Beijing, China; 2 Capital Medical University, Beijing, China

**Keywords:** baseline HbA1c, dose-response relationship, meta-analysis, renal function, SGLT2 inhibitors

## Abstract

**Background:**

Type 2 diabetes mellitus (T2DM) is a major global health challenge due to high cardiovascular risk. Sodium-glucose cotransporter 2 (SGLT2) inhibitors can offer glycemic and cardiorenal benefits. Most agents are available in low and high doses, with the assumption that higher doses improve glycemic control. However, previous evidence shows only marginal hemoglobin A1c (HbA1c) reduction (≈0.08–0.18%) with high doses, raising uncertainty about their clinical necessity. Patient factors such as baseline HbA1c and renal function influence SGLT2 efficacy, but whether these factors modify dose response remains unclear. This study evaluates dose-dependent effects across HbA1c and renal function strata.

**Objective:**

To assess the glycemic impact of high- versus low-dose SGLT2 inhibitors in T2DM, stratified by HbA1c and renal function.

**Methods:**

This analysis followed PRISMA guidelines (PROSPERO ID: CRD42024605351). PubMed, the Cochrane Library, and EMBASE were systematically searched for randomized controlled trials involving SGLT2 inhibitors in adults with T2DM through November 24, 2024. The primary outcome was change in glycated hemoglobin, stratified by hemoglobin A1c (HbA1c) and glomerular filtration rate (GFR) levels. Subgroup analyses were performed based on different SGLT2 inhibitors and dosages.

**Results:**

A total of 23 studies were included for the meta-analysis. Seventeen studies (n = 7,021) were stratified by HbA1c, and eight (n = 7,998) by GFR. Overall, high-dose SGLT2 inhibitors showed a slightly better glycemic control than low-dose SGLT2 inhibitors, with an additional 0.08% (95%CI: -0.12, -0.04) reduction in HbA1c levels. High-dose vs. low-dose SGLT2 inhibitors showed a 0.06%-0.16% further HbA1c reduction across varying glycemia levels (with HbA1c under or over 8%, 8.5%, 9%) and a change in HbA1c levels ranging from -0.07% to 0.04% across varying GFR levels (with GFR under or over 45, 60, 90 ml/min/1.73m^2^).

**Conclusion:**

Dose escalation had minimal effect on HbA1c across glycemic and renal strata; higher doses of SGLT2 inhibitors offer limited additional benefit for glycemic control in poorly controlled T2DM.

**Systematic Review Registration:**

https://www.crd.york.ac.uk/prospero/, identifier CRD42024605351.

## Introduction

Type 2 diabetes mellitus (T2DM) is a prevalent and complex chronic disease. Due to its association with cardiovascular-related morbidity and mortality, T2DM presents a significant global health challenge. The introduction of sodium-glucose cotransporter 2 (SGLT2) inhibitors, first approved in 2013, marked a novel therapeutic strategy for managing hyperglycemia in T2DM (1–3). These agents act by inhibiting renal tubular glucose reabsorption, thereby promoting urinary glucose excretion.

Nearly all SGLT2 inhibitors available on the market are prescribed in two dosage levels: a higher and a lower option. The lower dose is typically recommended as the starting regimen, with escalation to the higher dose for patients requiring further glycemic control. This recommendation is made under the assumption that higher doses offer superior blood glucose reduction than lower doses.

However, prior meta-analyses have reported that the differences in glycemic-lowering efficacy between high and low doses are minimal. Specifically, high-dose SGLT2 inhibitors reduce glycated hemoglobin (HbA1c) by approximately 0.08%–0.18% more than their low-dose counterparts (4–8). These findings raise an important clinical question: Is it necessary to prescribe higher doses for all patients with poor glycemic control, or are there particular subgroups that benefit more from dose escalation?

The baseline blood glucose levels and renal functions of patients significantly influence glucose-lowering outcomes (9). SGLT2 inhibitors demonstrate greater efficacy in patients with higher HbA1c levels than in those with lower levels (10, 11). Conversely, their potency is reduced in individuals with a decreased glomerular filtration rate (GFR) when compared to patients who have a GFR within the normal range (9). However, it remains unclear whether high-dose SGLT2 inhibitors provide additional benefit over low doses in patients with varying levels of glycemia and renal function. To date, no meta-analysis has specifically addressed this question.

Therefore, this study aimed to systematically evaluate the efficacy of all currently available SGLT2 inhibitors reported by randomized clinical trials (RCTs) at high and low doses in patients with T2DM, stratified by baseline HbA1c and renal function.

## Materials and methods

**Figure 1:**
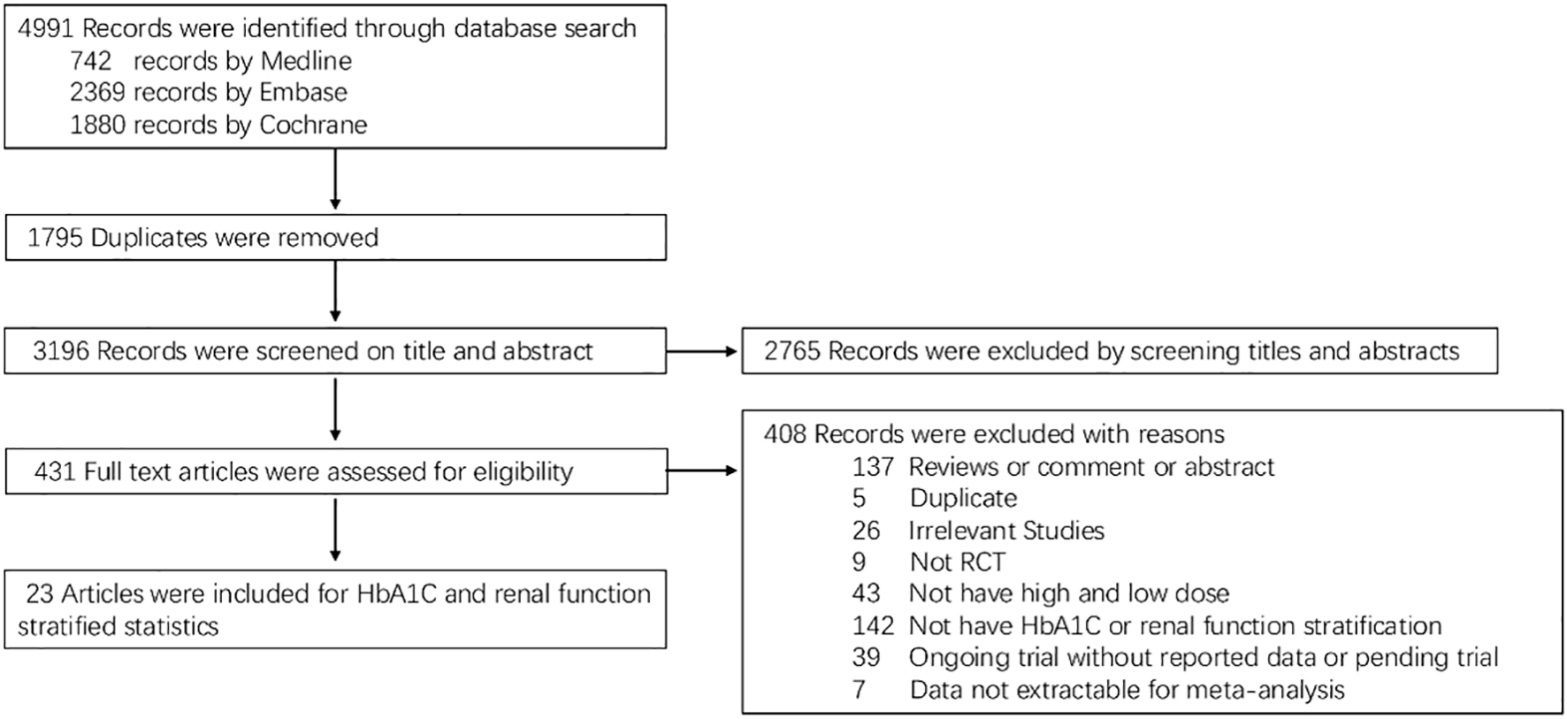
PRISMA flowchart depicting the process of selection of studies.

This systematic review and meta-analysis were conducted in accordance with the Preferred Reporting Items for Systematic Reviews and Meta-Analyses (PRISMA) Statement for traditional pairwise meta-analysis (PROSPERO ID: CRD42024605351) (28). The PRISMA flowchart for the selection of studies is presented in **Figure 1**, and the PRISMA checklist is shown in **Supplementary Table 1**.

### Study sources and searches

PubMed, Cochrane Library, and EMBASE were searched for RCTs of SGLT-2 inhibitors up to November 24, 2024. Two researchers (i.e., RX and YY) independently conducted literature searches with a specifically designed search strategy (**Supplementary Table 2**), later verified by another researcher (i.e., HZ).

### Study selection and outcomes

The current analysis encompassed studies that are RCTs targeting adult patients with T2DM. The intervention in these studies involved the use of SGLT2 inhibitors, either as a standalone therapy or in conjunction with other glucose-lowering treatments. The patients in each RCT received SGLT2 inhibitors at both the approved high and low dosages. The key outcome measure was the alteration in the glycated hemoglobin level. Each study had a follow-up period of at least 12 weeks, and the participant pool consisted of a minimum of 100 patients. The criteria for the exclusion of the studies included conference abstracts, reviews, letters, editorials, case reports, observation studies, and extension studies.

The study outcome was stratified in two aspects, as glycated hemoglobin level and renal function. Prespecified subgroup analyses were performed based on the consideration of different glycation levels (HbA1c ≥8% and HbA1c <8%; HbA1c ≥8.5% and HbA1c <8.5%; HbA1c ≥9% and HbA1c <9%) or kidney function levels (GFR ≥45 mL/min/1.73 m^2^ and GFR <45 mL/min/1.73 m^2^, GFR ≥60 mL/min/1.73 m^2^ and GFR <60 mL/min/1.73 m^2^, GFR ≥90 mL/min/1.73 m^2^ and GFR <90 mL/min/1.73 m^2^) and individual SGLT2 inhibitors (canagliflozin 100 mg and 300 mg; dapagliflozin 5 mg and 10 mg; empagliflozin 10 mg and 25 mg; ertugliflozin 5 mg and 15 mg; henagliflozin 5 mg and 10 mg; ipragliflozin 50 mg and 100 mg; luseogliflozin 2.5 mg and 5 mg; janagliflozin 25 mg and 50 mg; sotagliflozin 200 mg and 400 mg).

Two researchers (i.e., RX and YY) independently reviewed the titles and abstracts and assessed the full texts of the retrieved studies. Any disagreements arising during the analysis were resolved through consultation with the third researcher (i.e., HZ).

### Data extraction and quality assessment

Data were extracted from published articles, supplements, appendices, and public repositories that met the inclusion and exclusion criteria.

Two independent reviewers (i.e., RX and YY) performed the quality assessment using the Cochrane Collaboration’s tool (RoB 2.0). Considering the risk of bias, the following aspects were evaluated: randomization process, deviations from intended interventions, missing outcome data, the measurement of the outcome, the selection of the reported results, and other biases that could induce confounding effects.

### Data synthesis and analysis

For continuous variables, results were presented as mean differences (MD) along with 95% confidence intervals (CIs). The random-effects model, fitted by the inverse variance-weighting method, was employed to assess the overall estimated effects. Heterogeneity was analyzed using the I^2^ statistic, which measures the total variation between studies (significance for I^2^ > 50%) (Higgins and Thompson, 2002). Statistical significance was set at p < 0.05.

The influence of individual studies was examined through a leave-one-out sensitivity analysis. Continuous outcome changes (HbA1c% and GFR in mL/min/1.73 m^2^) with mean differences <0 were interpreted as favoring high-dose SGLT2 inhibitors compared with low-dose agents (95% CI, p < 0.05). Forest plots were created from the meta-analysis estimates using R software (v4.3.3) and Review Manager (v5.3), with statistical significance defined as a two-tailed p <0.05.

## Results

### Study selection and characteristics of studies included

A total of 5,023 articles were identified through database searches. After removing duplicates, 3196 articles were screened by title and abstract for eligibility. Of these, 431 articles underwent full-text review, and 23 studies met the inclusion criteria for stratified analysis based on HbA1c and renal function (**Figure 1**).

All 23 studies were included in traditional pairwise meta-analyses. Of these, 17 studies (n = 7,021) were included in the HbA1c-stratified analysis, while 8 studies (n = 7,998) were included in the GFR-stratified analysis. Detailed characteristics of the included studies and participants are presented in **Table 1**.

**Table 1:** Baseline characteristics of included studies.

No.	Author (Year)	Registration number	Background therapy	Con	Drug	Total sample size	Sample size of low dose SGLT2i	Sample size of high dose SGLT2i	Mean age (Y)	Sex (male%)	Mean BMI (kg/m^2^)	HbA1c (%)	Mean HbA1c (%)	Mean duration of diabetes (year)	Race (primary)	Country (mainly)		Follow-up duration (weeks)
1	Wilding, JP (2015)	NCT01081834 NCT01106677 NCT01106625 NCT01106690	No AHA, MET, MET+SU, MET+PIO	PLA	CAN	2313	833	834	55.9	49	32.1	7.0-10.5	8	7.3	White	United Kingdom		26
2	Yamout, H (2014)	NCT01106651 NCT01081834 NCT01032629	DE AHA	PLA	CAN	1085	338	365	67.1	59	32.3	7.0-10.5	8.1	15.2	White	USA		18-26
3	Ji, L (2014)	NCT01095653	NR	PLA	DAP	393	128	133	52.1	65.2	25.5	7.5-10.5	8.2	0.3	Chinese	China		24
4	Kaku, K (2014)	NR	DE	PLA	DAP	261	86	88	58.1	59.2	25.5	6.5-10.0	7.5	4.8	Japanese	Japan		24
5	Kohan, DE (2014)	NCT00663260	DE±AHA	PLA	DAP	252	83	85	67	66.1	NR	7.0-11.0	8.3	18	White	USA		104
6	Strojek, K (2011)	NCT00680745	GLI	PLA	DAP	592	142	151	59.7	47.9	NR	7.0-10.0	8.1	7.5	Europe	Polen		24
7	DeFronzo, RA (2015)	NCT01422876	MET	LINA	EMP	674	135	134	56.2	54.6	31	7.0-10.5	8	NR	White	USA		52
8	Haring, HU (2013)	NCT01159600	MET+SU	PLA	EMP	666	225	216	57.1	51	28.2	7.0-10.0	8.1	NR	Asia	Germany		24
9	Inzucchi, SE (2021)	NCT01159600	DE+MET	PLA	EMP	637	217	213	55.6	57.5	29.4	7.0-10.0	7.9	NR	White	Germany		24
10	Ji, L (2023A)	NCT04233801	INS±-OADs	PLA	EMP	219	73	73	60.2	54.3	25.9	7.5-11.0	8.6	14.6	Chinese	China		24
11	Kadowaki, T (2014)	NCT01193218	DE AHA	PLA	EMP	547	109	109	57.2	75.3	25.4	7.0-10.0	8.0	NR	Japanese	Japan		12
12	Roden, M (2013)	NCT01177813	No AHA	PLA	EMP	899	224	224	55.0	64	28.3	7.0-10.0	7.9	NR	Asia	China		24
13	Sone, H (2020)	NCT02589639	INS	PLA	EMP	269	89	90	58.5	70.6	26.9	7.0-10.0	8.8	15	Japanese	Japan		16
14	Wanner, C (2018)	NCT01131676	No AHA AHA	PLA	EMP	7018	2345	2340	63.1	71.4	30.6	7.0-10.0	8.1	NR	White	Europe		12
15	Dagogo-Jack, S (2021)	NCT01986881	No AHA	PLA	ERT	1769	615	557	68.1	64	32.4	7.0-10.5	8.2	15.9	White	USA		28
16	Hollander, P (2018)	NCT01999218	MET	GLI	ERT	1325	448	440	58.4	47.1	31.5	7.0-9.0	7.8	7.5	White	USA		52
17	Liu, J (2019)	NCT01958671 NCT02033889 NCT02036515	No AHA, MET, MET+SIT	PLA	ERT	1543	519	509	57.3	52.6	31.5	7.0-10.5	8.1	7.5	White	North America		26
18	Pratley, R.E (2018)	NCT02099110	MET	SIT	ERT	1233	236	241	55.2	51.8	31.9	7.5-11.0	8.6	7.1	White	USA		26
19	Weng, J (2021)	NCT04390295	MET	PLA	HEN	483	162	160	54.7	63	25.5	7.5-11.0	8.5	5.9	Chinese	China		24
20	Kashiwagi, A (2014)	NCT00621868	No AHA	PLA	IPR	361	71	72	57	63	26	7.4-10.5	8.3	1.5	Japanese	Japan		12
21	Ji, L (2023B)	NCT03811548	DE	PLA	JAN	432	142	142	52	65	26	7.0-10.5	8.4	NR	Chinese	China		24
22	Seino, Y (2014)	JapicCTI-101191	No AHA	PLA	LUS	282	56	54	57.6	64.8	24.7	6.9-10.5	8.1	6	Japanese	Japan		12
23	Cherney, DZI (2023)	NCT03242252	INS OADs	PLA	SOT	787	263	264	69.5	56.4	32.4	7.0-11.0	8.3	17.1	White	Canada		26

Con, Control; SGLT2i, Sodium glucose transporter 2 inhibitors; CAN, Canagliflozin; DAP, Dapagliflozin; EMP, Empagliflozin; IPR, Ipragliflozin; LUS, Luseogliflozin; ERT, Ertugliflozin; HEN, Henagliflozin; JAN, Janagliflozin; SOT, Sotagliflozin; AHA, Anti-hyperglycemic agents; OAD, Oral antidiabetic drugs; DE, Diet and exercises; MET, Metformin; INS, Insulin; SU, Sulfonylureas; SIT, Sitagliptin; PIO, Pioglitazone; LINA, Linagliptin; GLI, Glimepiride; NR, not reported. USA, united states of American.

### Risk of bias and quality of evidence

Risk of bias assessments are provided in **Supplementary Table 3** and **Supplementary Figure 1**. All included trials had either low risk or some concerns across the five evaluated domains. The overall risk of bias for outcomes in the pairwise meta-analyses was assessed as low to moderate in comparisons between high-dose and low-dose SGLT2 inhibitors.

### Overall and stratified subgroup analysis on the effects of SGLT2 inhibitors with high dose and low dose on glycemic control

**Figure 2:**
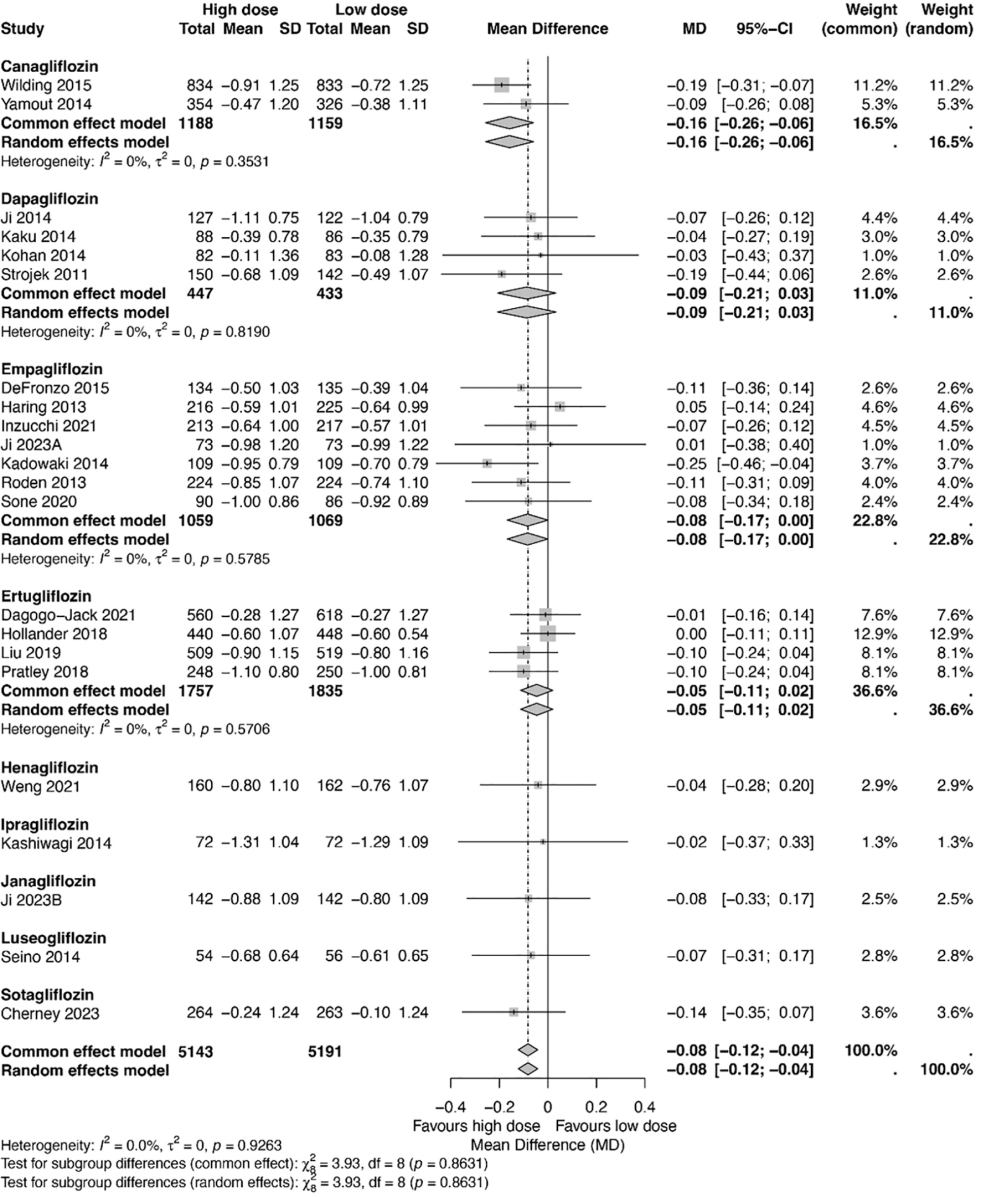
A forest plot showcasing differences in the glucose-lowering effects of high-dose and low-dose SGLT2 inhibitors, stratified by drug classification. Total, numbers of studies; Mean, mean difference; SD, standard deviation; CI, confidence interval; I^2^, heterogeneity.

**Figure 2** presents all studies included in this meta-analysis that reported both high- and low-dose treatment arms. High-dose SGLT2 inhibitors were modestly more effective in lowering blood glucose levels compared to low-dose formulations, with a mean difference of −0.08% (95% CI: −0.12, −0.04). Among the agents evaluated, canagliflozin showed the greatest effect (−0.16%), followed by dapagliflozin (−0.09%), empagliflozin (−0.08%), and ertugliflozin (−0.05%). No significant heterogeneity was observed across the studies (p = 0.93).

**Figure 3:**
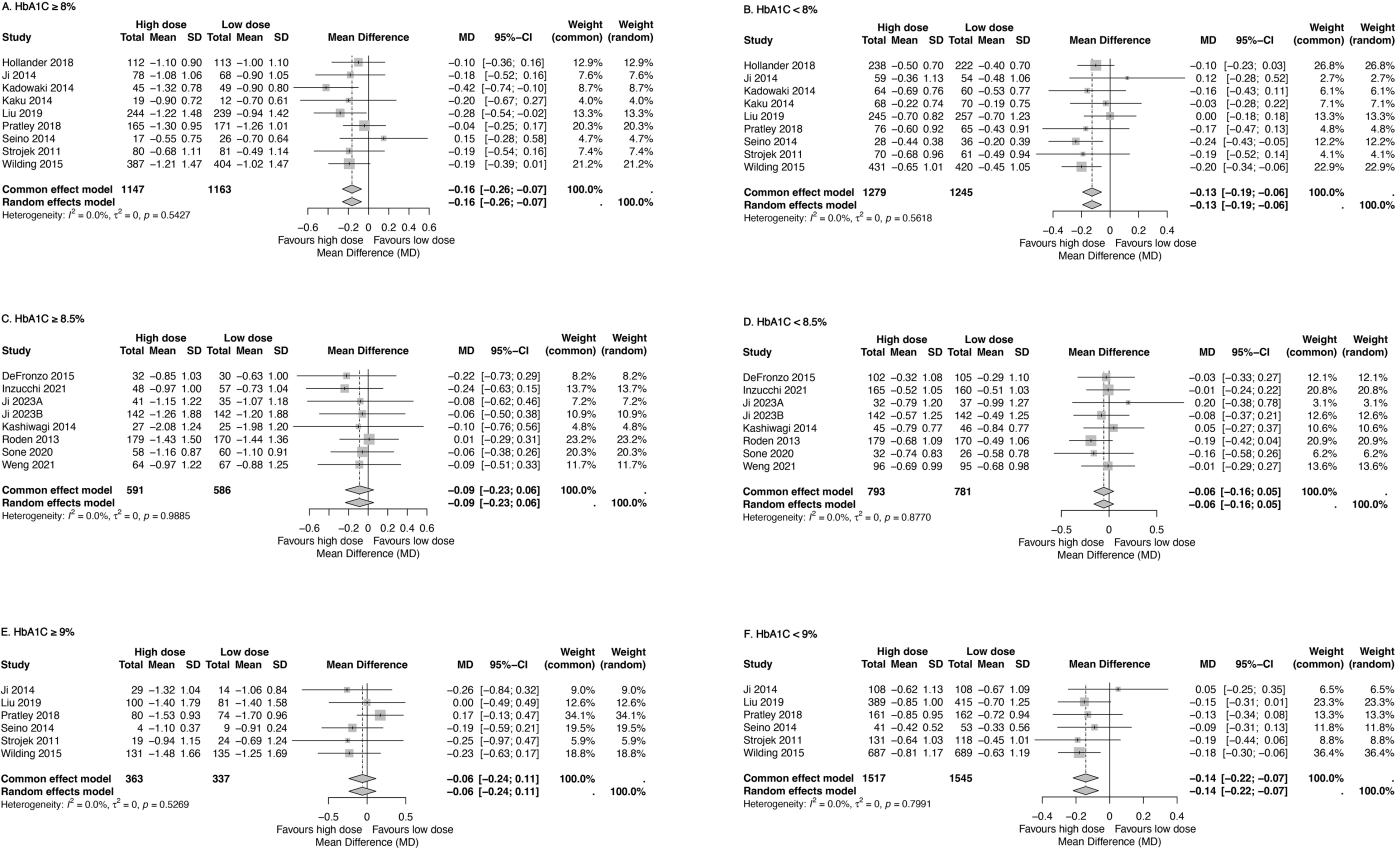
Forest plots displaying the impacts of high-dose and low-dose SGLT2 inhibitors on the mean reduction of HbA1c level compared among patients with HbA1c ≥8% **(A)** or <8% **(B)**, ≥8.5% **(C)**, or <8.5% **(D)**, ≥9% **(E)**, and <9% **(F)**. Total, numbers of studies; Mean, mean difference; SD, standard deviation; CI, confidence interval; I^2^, heterogeneity.

The effect of dosage on HbA1c reduction was further evaluated across different glycemic strata: HbA1c above or below 8%, 8.5%, and 9%. Across all comparisons, high-dose SGLT2 inhibitors reduced HbA1c by an additional 0.06% to 0.16% compared with low-dose treatments (**Figure 3**).

**Figure 4:**
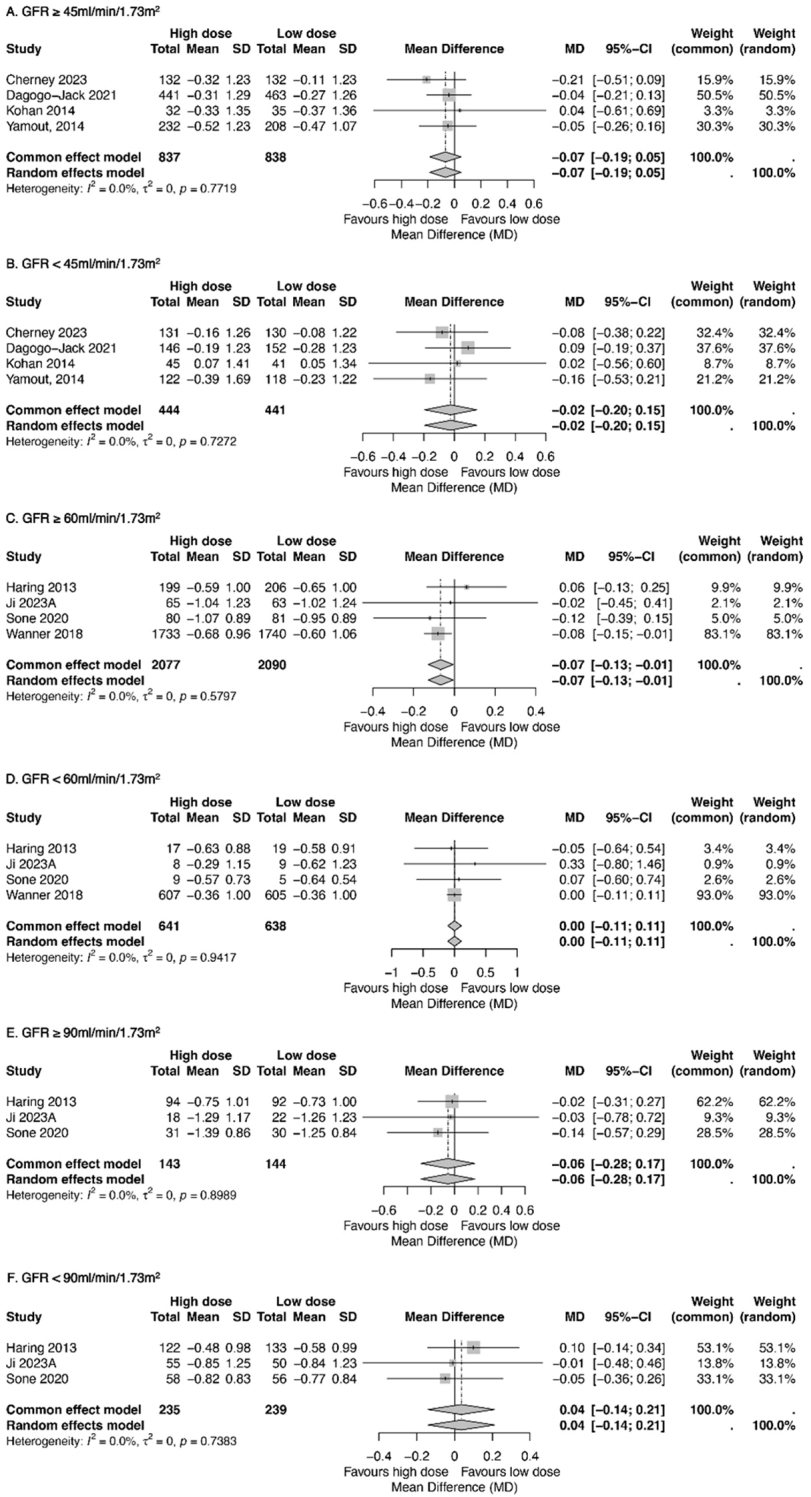
Forest plots exhibiting the impacts of high-dose and low-dose SGLT2 inhibitors on the mean reduction of the HbA1c level compared among patients with GFR ≥45 mL/min/1.73 m^2^**(A)** or <45 mL/min/1.73 m^2^**(B)**, patients with GFR ≥60 mL/min/1.73 m^2^**(C)** or <60 mL/min/1.73 m^2^**(D)**, and patients with GFR ≥90 mL/min/1.73 m^2^**(E)** or <90 mL/min/1.73 m^2^**(F)**. Total, numbers of studies; Mean, mean difference; SD, standard deviation; CI, confidence interval; I^2^, heterogeneity.

The glycemic effects of high- and low-dose SGLT2 inhibitors were also analyzed by renal function, comparing participants with GFR above or below 45, 60, and 90 mL/min/1.73m^2^. The results showed minimal differences in HbA1c reduction between dosage groups across all renal function strata, with changes ranging from −0.07% to 0.04% (**Figure 4**).

**Figure 5:**
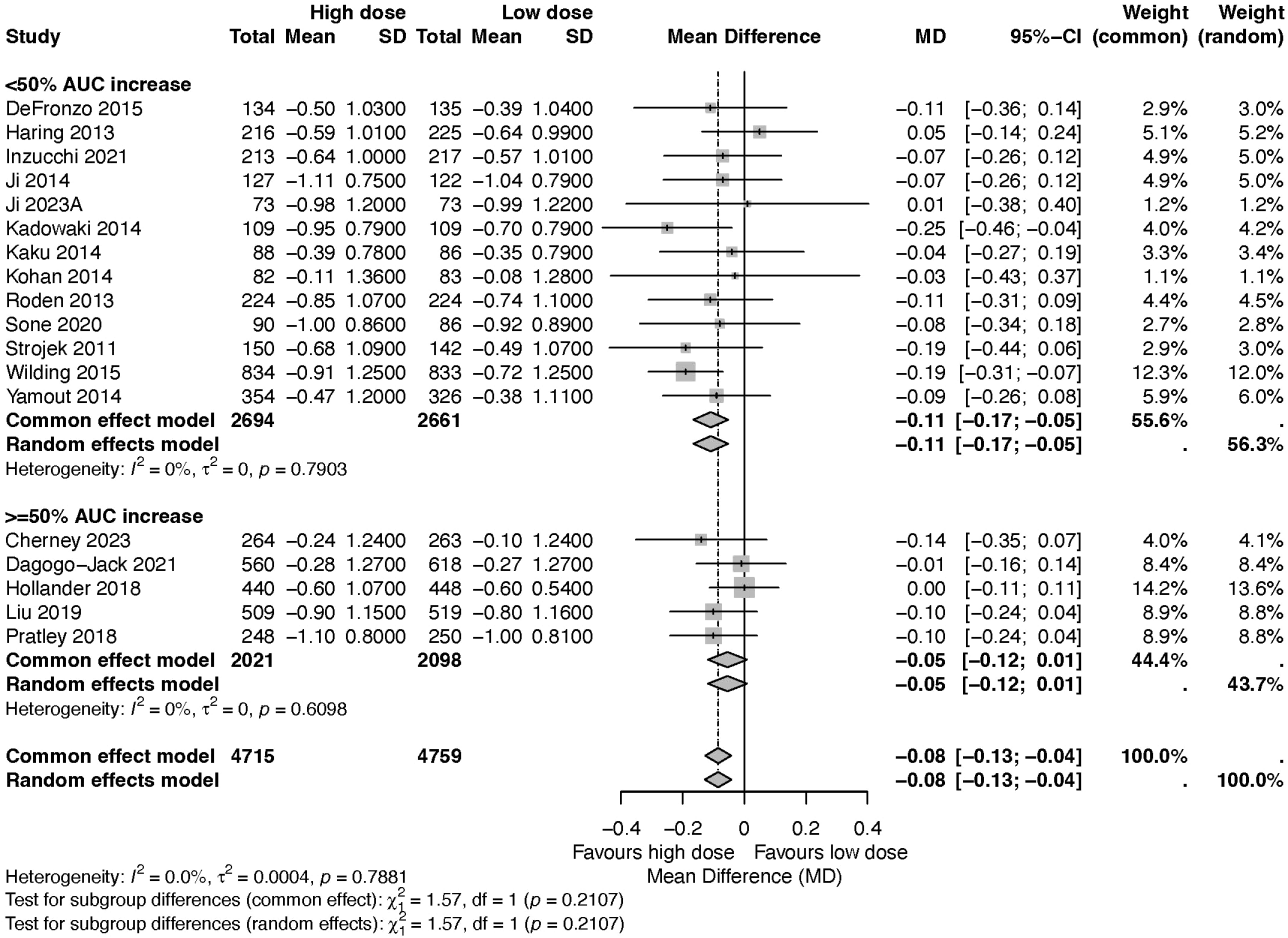
A forest plot showing the differences in the glucose-lowering effects of high-dose versus low-dose SGLT2 inhibitors, stratified by AUC increase of drug exposure.

Considering concerns that intrinsic differences among individual SGLT2 inhibitors, such as affinity, potency, metabolic disposition, and pharmacokinetic and pharmacodynamic profiles could influence the observed differences between high-dose vs low-dose regimens, we performed an additional stratified subgroup meta-analysis based on the magnitude of AUC increase of the drug exposure in mild renal impairment (<50% vs ≥50%). Results indicates that high-dose SGLT2 inhibitors produced a modest additional HbA1c reduction compared with low-dose therapy (MD −0.08%, 95% CI −0.13 to −0.04), with no evidence of heterogeneity (I^2^ = 0.0%). In the <50% AUC increase subgroup, the dose-related HbA1c difference was larger and statistically significant (MD −0.11%, 95% CI −0.17 to −0.05; I^2^ = 0%). In the ≥50% AUC increase subgroup, the effect was smaller and not statistically significant (MD −0.05%, 95% CI −0.12 to 0.01; I^2^ = 0%). The test for subgroup differences was not significant (p = 0.2107) (**Figure 5**).

Overall heterogeneity among study results was low, and no significant heterogeneity was detected in the analyses.

### Sensitive analyses

A leave-one-out sensitivity analysis was conducted by sequentially removing each study to assess its influence on the overall findings. The results remained stable regardless of which study was excluded, indicating the robustness of the conclusions (**Supplementary Figure 2**).

### Publication bias

No evidence of publication bias was detected. Funnel plots from the traditional pairwise meta-analysis were visually symmetrical, supporting the absence of bias (**Supplementary Figure 3**). No heterogeneity was found in Baujat plot and Galbraith plot (**Supplementary Figure 4**).

## Discussion

### Major findings and interpretations

This systematic review and meta-analysis assessed the overall efficacy of high- versus low-dose SGLT2 inhibitors in patients with varying HbA1c and GFR levels, based on 23 RCTs.

Overall, our findings indicate that high-dose SGLT2 inhibitors offered only a modest glycemic advantage over low-dose formulations, with an additional HbA1c reduction of just 0.06%–0.16% across different glycemic strata. The effect varied minimally across renal function groups, with differences ranging from −0.07% to 0.04%. Notably, the consistently small differences observed also address a key concern regarding pharmacokinetic (PK) heterogeneity in renal impairment. Using prior PK summaries that reported drug-specific increases in AUC with declining kidney function, we conducted a subgroup analysis stratified by AUC, in which SGLT2 inhibitors were classified according to the magnitude of AUC increase in mild renal impairment (26, 27). This classification was directly based on the AUC data compiled in those earlier reports. While point estimates suggested a somewhat larger difference among agents with <50 % AUC increase compared to those with ≥50 % increase, the interaction test was non-significant, indicating that this categorization did not robustly modify the treatment effect in our dataset. Together, our results suggest that any dose–response differences are unlikely to be meaningfully driven by PK heterogeneity related to renal impairment, at least within the available evidence.

Previous studies also reported slight efficacy differences between high and low doses of SGLT2 inhibitors. However, unlike earlier work, our study is the first to stratify results by both HbA1c and GFR levels and to include a broader range of agents, including recently approved drugs such as henagliflozin, janagliflozin, and sotagliflozin^5,8^. By comparing different HbA1c and GFR subgroups, we found only minor differences in glycemic effect between high- and low-dose SGLT2 inhibitors—even among patients with HbA1c greater than 9% and GFR greater than 90 mL/min, who might theoretically benefit more from higher doses. These results suggest that the added glycemic benefit of higher doses may be clinically limited.

### Mechanisms for findings

The minimal difference in glucose-lowering efficacy between high and low doses likely reflects that urinary glucose excretion does not exhibit a strong dose–response relationship for SGLT2 inhibitors. For example, in three independent studies, the mean 24-h urinary glucose excretion in the 10 mg and 25 mg empagliflozin groups was 81 g and 93 g, 50 g and 58 g, and 64 g and 78 g, respectively. Similarly, in the 100 mg and 300 mg canagliflozin groups, it was 99 g and 95 g, and in the 5 mg and 10 mg dapagliflozin groups, it was 44 g and 54 g (12–14).

Although renal glucose excretion increases with higher GFR and plasma glucose levels (9), no studies to date have evaluated how different SGLT2 inhibitor doses influence glucose excretion in patients with varying HbA1c and GFR levels. Our analysis showed that even in patients with elevated HbA1c, the difference in glycemic reduction between high and low doses was minimal—comparable to that observed in patients with lower HbA1c levels. This suggests that both high and low doses have a similar impact on renal glucose excretion regardless of baseline glycemia. Moreover, since reduced GFR impairs urinary glucose excretion, the lack of efficacy difference between high and low doses across GFR categories further supports the notion that increasing SGLT2 inhibitor dosage may offer limited additional benefit in such patients.

### Current evidence on the impact of SGLT2 inhibitor dosages on weight loss, blood pressure control, cardiorenal outcomes, and adverse effects

In addition to their glucose-lowering properties, SGLT2 inhibitors provide benefits related to weight loss, blood pressure reduction, and cardiorenal protection. However, these clinical effects do not appear to differ substantially between high and low doses. Meta-analyses have shown that high-dose SGLT2 inhibitors confer a marginally greater reduction in body weight (approximately 0.263–0.556 kg) than low-dose regimens (8, 15, 16). In contrast, no significant advantage has been observed with higher doses in terms of additional blood pressure reduction (17, 18).

Current evidence on dose-dependent differences in the cardioprotective effects of SGLT2 inhibitors remains limited. A network meta-analysis reported no significant difference in the reduction of all-cause mortality and cardiovascular events across varying doses of SGLT2 inhibitors (19). Similarly, nephroprotective outcomes—including changes in albuminuria and estimated glomerular filtration rate (eGFR)—showed no notable variation between high- and low-dose regimens of dapagliflozin and empagliflozin (20, 21).

SGLT2 inhibitors are associated with several known adverse effects, including diabetic ketoacidosis, urinary tract and genital infections, hypotension, and less common outcomes such as amputations and acute kidney injury. Several meta-analyses have reported no significant difference in the risk of these adverse events between high and low doses (22, 23). One meta-analysis showed that the incidence of any adverse event was 69.4% (8,417/12,136) in the high-dose group and 69.3% (8,383/12,089) in the low-dose group (22). Nevertheless, some data suggest a potential increase in specific side effects with higher doses. For example, dapagliflozin 10 mg daily was significantly associated with an increased risk of urinary tract infections, whereas dapagliflozin 5 mg was not (24, 25). A trend toward more volume-related adverse events was also observed with canagliflozin 300 mg compared with 100 mg (relative risk: 1.35; 95% CI: 0.99–1.85; P = 0.059) (22).

In conclusion, there appear to be minimal or no significant differences in weight loss, blood pressure control, cardiovascular outcomes, or renal outcomes between high- and low-dose SGLT2 inhibitors. Given the potential for increased side effects and higher treatment costs associated with larger doses, careful evaluation of the benefit–risk balance is warranted when considering higher-dose regimens.

### Strengths and limitations

Our study conducted a comprehensive evaluation of the glycemic effects of varying dosages of SGLT2 inhibitors in patients with T2DM, stratified by baseline HbA1c levels and renal function. In contrast to a similar meta-analysis published in 2021, the present study incorporated 15 additional RCTs. Moreover, this meta-analysis is the first to introduce subgroup analyses based on glycemic status and renal function—an analytical dimension not previously explored in earlier studies. Additionally, we examined a wider spectrum of SGLT2 inhibitors and found only limited differences in glucose-lowering efficacy between the currently prescribed high and low clinical doses. These findings enrich the current understanding of dose-response relationships for SGLT2 inhibitors and support more evidence-based clinical decision-making.

Despite its strengths, this meta-analysis has several limitations. First, most of the included trials excluded participants with an estimated GFR below 30 mL/min/1.73 m^2^, thereby limiting our ability to assess treatment effects in patients with stage 4 chronic kidney disease (CKD). Second, the relatively small number of studies available for specific outcomes—particularly those stratified by renal function—restricted our capacity to conduct more granular subgroup analyses, including those based on sex, age, and comorbidities.

## Conclusions

In summary, our findings suggest that dosage variation among SGLT2 inhibitors does not result in clinically meaningful differences in glycemic improvement across patient groups with varying glucose levels or diverse renal function statuses. Therefore, for individuals with poorly controlled T2DM, initiating or escalating to high-dose SGLT2 inhibitor therapy may not confer additional glycemic benefit. When considering higher dosages, clinicians should carefully evaluate the benefit-risk ratio to ensure optimal therapeutic outcomes.

## Data Availability

The original contributions presented in the study are included in the article/**Supplementary Material**. Further inquiries can be directed to the corresponding authors.

## Supplementary material

Supplementary Table 1 PRISMA Checklist.

Supplementary Table 2 Electronic search strategies (Search date: November 24th, 2024).

Supplementary Table 3 Quality assessment results of included randomized controlled trials.

Supplementary Figure 1 Quality assessment results of included randomized controlled trials.

Supplementary Figure 2 Sensitivity analysis using leave-one-out test.

Supplementary Figure 3 Assessment of publication bias of the studies using Funnel plots.

Supplementary Figure 4 Heterogeneity test using Baujat plot and Galbraith plot.
